# Task-related attentional processes are distinctly modulated by respiration and RR interval variability

**DOI:** 10.1016/j.jphyss.2025.100053

**Published:** 2025-12-22

**Authors:** Nozomu H. Nakamura, Kohzoh Yoshino, Masaki Fukunaga

**Affiliations:** a Department of Integrative Physiology, Faculty of Medicine, Hyogo Medical University, 1-1, Mukogawa cho, Nishinomiya, , Hyogo 663-8501, Japan; b Department of Biomedical Sciences, School of Biological and Environmental Sciences, Kwansei Gakuin University, 1, Gakuen Uegahara, Sanda, , Hyogo 669-1330, Japan; c Section of Brain Function Information, National Institute for Physiological Sciences, 38 Nishigonaka Myodaiji, Okazaki, Aichi 444-8585, Japan; d Graduate Institute for Advanced Studies, SOKENDAI, Hayama, Kanagawa 340-0193, Japan

**Keywords:** Cardiorespiratory coupling, Cognitive processes, Response times, Interoception, Respiratory sinus arrhythmia

## Abstract

Changes in reaction times (RTs) during attentional processing may be associated with the state of breathing. Although breathing and cardiac activity interact, the functional importance of the cardiorespiratory system for modulating attentional processing remains unclear. To determine the involvement of respiration and RR interval (RRI) variability in successful task performance, thirty-six healthy participants performed a short-term memory task. For RTs limited to correct responses, increases were observed under two conditions: i) when inspiratory onset (or exhalation-to-inhalation transition) occurred and ii) when RRI velocity increased during retrieval. Importantly, multilevel model analysis revealed that the timing of inspiratory onset and the increase in RRI velocity were temporally mismatched, suggesting that at least two independent mechanisms may prolong RT. These findings contribute to a better understanding of how respiration and RRI variability may be functionally differentiated and how each could be linked to attentional processes that influence performance.

## Highlights

• RT limited to successful task performance increased when inspiratory onset occurred.
• RT limited to successful task performance also increased when the RRI velocity increased.
• The occurrence of inspiratory onset and RRI fluctuations were temporally separated.
• They may modulate successful task performance through distinct attentional processes.

## Background

When we encounter a person, we immediately determine whether we know that person. This determination is a real-world example of a fundamental cognitive process. In this process, signal detection theory can aid in accurate discrimination (e.g., between a friend and a stranger) in the presence of uncertainty [1]. Discriminability is estimated by separation between distributions of “old” and “new” items not only during long-term memory tasks [2], [3] but also during short-term memory tasks [4], [5]. However, uncertainty often obscures separation and interrupts successful task performance.

One potential factor that limits successful task performance is attentional processing [6]. Attentional processes are closely associated with changes in reaction time (RT), which refers to the temporal duration between the presentation of a sensory cue and a button-press action. Each RT in the task fluctuates across a variety of external and internal states. Specifically, a shorter RT often indicates immature responses and an absence of awareness of the potential need for response inhibition [7]. In contrast, a longer RT may be associated with reduced attention to the stimulus with a brief interruption of top-down signals [8]. Esterman et al. [9] reported that RT task trials with longer RTs presented higher failure rates than those with shorter RTs did. Critically, a longer RT in conjunction with correct responses induced activation in the dorsal attention network (DAN) and parahippocampal regions compared with a longer RT in conjunction with failed responses, whereas a shorter RT in conjunction with correct responses induced deactivation in the default-mode network [9]. Even though a button-press response or motor action is another factor that affects RT during a task, attentional processing in the brain appears to play a pivotal role in successful performance.

Recent findings have demonstrated that task performance (i.e., RT and accuracy) is modulated by the timing and phases of the respiratory cycle [10], [11], [12]. Physiologically, respiration is composed of three distinct phases––inspiration, postinspiration, and active expiration––coordinated by the pontomedullary network [13], [14], [15], [16]. Notably, the primary respiratory rhythm generator, the preBötzinger complex (PreBötC) in the ventrolateral medulla initiates its firing at inspiratory onset, corresponding to the exhalation-to-inhalation (EI) transition. Accumulating evidence indicates that these phases alter task performance. In a visuospatial task, accuracy was greater when the presentation of test cues coincided with the EI transition (inspiratory onset) than when it coincided with the inhalation-to-exhalation (IE) transition (postinspiratory onset) [17]. Conversely, RT was prolonged and accuracy was reduced when the EI transition occurred in the middle of sensory detection [18] or retrieval of a recognition memory task [19]. Importantly, our recent study revealed that a specific retrieval process encompassing the EI transition is associated with decreased activity in the ventral attention network (VAN) compared with that associated with the IE transition [20]. Together, these findings suggest that the occurrence of EI transition during retrieval imposes attentional cost, leading to longer RT and potentially reduced performance [20], [21].

Attentional processing is increasingly recognized as being differentially shaped by a variety of interoceptive signals, such as heart rate, blood pressure, respiration, and intestinal peristalsis. This diversity arises because interoceptive pathways conveying these signals to the brain are multifaceted, involving both neural and hormonal regulatory mechanisms [22], [23]. Most visceral afferent signals are sent to the nucleus tractus solitarius (NTS) and insular cortex in the CNS [22], [23], whereas heart rate information has recently been reported to be transmitted to the olfactory bulb [24]. Interestingly, signals generated by the respiratory center in the pontomedullary network project to several candidate hubs [21], including the olfactory bulb [25], [26], parafacial nucleus, parabrachial nucleus [27], [28], locus coeruleus [29], and thalamus [30]. Accordingly, these diverse projection pathways suggest that interoceptive signals may engage functionally distinct mechanisms of attentional processing, although this possibility remains poorly delineated.

Respiratory cycles give rise to variations in heart rates, such as respiratory sinus arrhythmia (RSA), which are rhythmic fluctuations in the R-wave to R-wave interval (RRI) between heartbeats in accordance with inhalation and exhalation [31], [32], [33]. RSA or RRI variability is considered a critical index of the cardiovascular system and is induced by respiratory oscillations that may reflect attentional processes [34], [35]. Such cardiorespiratory coupling affects the efficiency of gas exchange and might act as a coupled oscillator to maintain rhythms [36]. However, the functional relevance of the association between respiration and RRI variability in attentional processing remains unclear. A critical question that arises is whether RRI variability directly modulates attentional processing during the retrieval phase of a cognitive task. If so, does RRI variability modulate RTs in the same way as, or in a different manner from, respiration? To address these questions, we investigated both respiratory phases and intraindividual fluctuations (i.e., velocity) in RRIs [37] and determined their effects on RT [9] during a short-term memory version of the delayed matching-to-sample (DMTS) task [19], [20]. Although memory encoding can reduce accuracy in the DMTS task, the present study focused on RTs limited to correct responses (i.e., correct RT), which reflect attentional processing during retrieval. These findings demonstrate potentially causal effects of visceral responses that may explain the distinct attentional mechanisms modulated by respiratory phases and RRI variability.

## Methods

### Subjects

The participants in this study were 39 healthy right-handed volunteers. All the subjects had abstained from caffeine-containing beverages for at least 12 h before the experiment. None of the subjects were regularly taking medication, and none had known histories of respiratory, cardiovascular, endocrine, neurological, or psychiatric diseases. Written informed consent was obtained from all participants. In three volunteers, no EI transition occurred during the test trials. Because EI-transition-dependent effects could not be evaluated in these individuals, they were excluded. In total, 36 healthy subjects (21 males and 15 females; age: 23.4 ± 0.6 years; range: 20–31 years) were included for further analysis. All procedures performed on humans were in accordance with the Declaration of Helsinki (Ethical Principles for Medical Research Involving Human Subjects) and Ethical Guidelines for Medical and Health Research Involving Human Subjects, Japan and were approved by the Ethics Committee of Hyogo College of Medicine, Japan (No. 1825).

### Apparatus

Inhalation and exhalation during the respiratory cycle were continuously recorded via a flow sensor nasal cannula (Flow Nasal Cannula A, 1 m, Atom Medical, Japan) equipped with a differential pressure transmitter (Model KL17, Nagano Keiki, Japan) [19]. An electrocardiogram (ECG) was recorded via lead II using a differential biological amplifier (Bioamp, AD Instruments, Dunedin, New Zealand). Respiratory waveforms, ECGs, and trigger signals during the experiment were sampled at 1 kHz using the PowerLab data acquisition system (PowerLab, AD Instruments) and processed online using LabChart software (LabChart 7.1, AD Instruments). Before each experiment, the air pressure in the room was measured to determine a baseline level, which was subsequently subtracted from raw respiratory waveforms. No additional filtering or artifact handling was applied to the respiratory data. Raw ECG traces were bandpass filtered (0.5–40 Hz) to remove baseline drift and high-frequency noise. R waves were automatically detected using the module’s QRS detection algorithm, followed by manual verification.

### Task paradigm

**Fig. 1:**
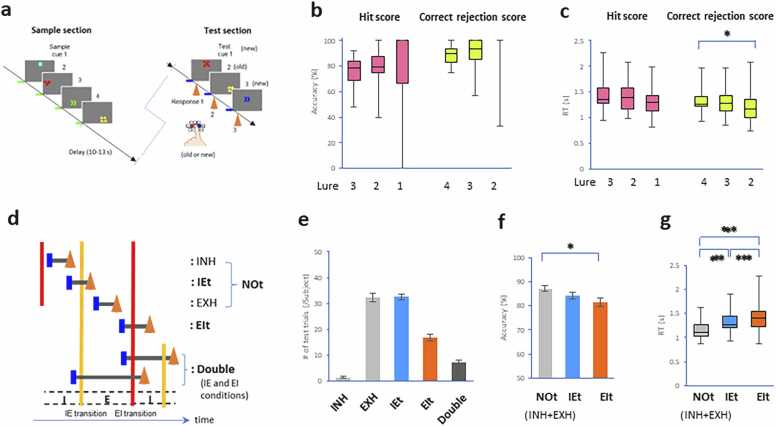
**DMTS task block, its accuracy, and its RT. a**. A task block consisting of four sample cues (green lines), a delay, and three test cues (blue line). The button press (orange arrows) was based on the observed decisions. **b,c**. Plots showing lure-dependent accuracy (**b**) and lure-dependent reaction time (RT, **c**) of the hit score (hit/hit + miss) and correct rejection score (correct rejection/correct rejection + false alarm) because each test trial contained different degrees of lures. **d**. Drawing showing conditions of combinations between test trials and phases of breathing: (i) test trials within inhalation (INH condition); (ii) test trials encompassing the inspiratory onset or IE transition (IEt condition); (iii) test trials within exhalation between postinspiration and active expiration (EXH condition); (iv) test trials encompassing the postinspiratory onset or EI transition (EIt condition); and (v) test trials encompassing both IE and EI transitions (double condition). The INH and EXH conditions were combined into the NOt condition. **f,g**. Plots showing the accuracy (**f**) and RT (**g**). Compared with any other condition, the EIt condition resulted in a longer RT. * *p* < 0.05, *** p < 0.005 (post hoc comparisons).

A DMTS version of a visual recognition memory task with short delay was employed as previously described [19], [20] with modifications. Each participant performed three blocks of the DMTS task, which consisted of a sample section, delay, and test section structured according to a standard DMTS protocol (Fig. 1a). Task paradigms were created in NBS Presentation software (Presentation 18.3, Neurobehavioral Systems, Berkely, CA). The DMTS task requires the memorization and recognition of visual images comprising a symbol (configuration), its color, its position, and the number of instances of the symbols on a computer screen (47.7 ×26.8 cm, 1920 ×1080 resolution, and a 60 Hz refresh rate). The symbol (configuration) was a circle, triangle, rectangle, cross, crescent, or heart; its color was red, blue, green, yellow, pink, or sky blue; the number of symbols was one, two, three, four, five, or six; and the symbol(s) were positioned at the center, right, left, top center, bottom right, or bottom left of the screen. Thus, there were 1296 (6 ×6 ×6×6 variables) possible combinations. Thus, the image processing speed varied in a randomized manner because of perceptual combination.

During the sample section, each volunteer was exposed to a series of four out of a total of 1296 images displayed one at a time on a computer screen under dim light in a darkened room. After a short delay (10–13 s), each volunteer was tested three separate times for the ability to distinguish between the image presented during the sample section (“old” or match image) and “new (or nonmatch) images selected from the 1292 out of 1296 images that were not presented during the sample section (Fig. 1a). After each test image was presented, the volunteer was required to indicate whether the presented image was the same as one of the four sample images presented and then to press the corresponding button. An “old” (or match) image was defined as an image that matched one of the sample images in all four characteristics: symbol (configuration), color, position, and number of figures. A “new” (or nonmatch) image was defined as an image that did not perfectly match any of the sample images in this manner. The volunteer pressed one of two buttons using their left fingers once they had identified the image shown during the test section as an “old” or “new” image.

Each volunteer memorized 4 sample images and recognized 3 test images per task block. Individual participants performed 30 blocks, in which a total of 90 test images were categorized as either old or new. In total, 45 “old” and 45 “new” images were presented randomly during the test. Before the experiments, the volunteers were instructed on how to perform the task and were told to breathe in a relaxed, natural manner during the task. They were not informed of the timing of the test figures during the task. We confirmed that participants did not control their breathing intentionally during the task, as no participants reported awareness of the figures being locked to their own respiratory cycles or of intentionally adjusting their breathing to correspond with the timing of figure exposure to improve their performance.

### Data analysis

During the experiments, we measured the following parameters: 1) cognitive parameters, including the time of figure exposure, time of button pressing, and accuracy as captured by NBS Presentation software; and 2) cardiorespiratory parameters, including the RRI and the onset of every inhalation and exhalation in the raw respiratory waveform, as captured by LabChart software. In the present study, the interval from an R wave (R_n_) to the previous R wave (R_n-1_) was defined as the RRI (Fig. 3a). The RRI velocity (ΔRRI) during the task was then defined by the subtraction between RRIs overlapping a test cue presentation and a button-press response (Fig. 3e). Since speed represents a strictly positive quantity and often exhibits a log-normal distribution across trials, analyses were performed in logarithmic space. The log transformation linearizes multiplicative relationships (e.g., twofold differences) and reduces heteroscedasticity, providing more appropriate scaling for comparing conditions with different absolute velocities.

At a preset level, the onset of every inhalation and exhalation was defined as the time at which the flow first crossed the baseline level and deviated from it by more than ± 2 SD to ensure that the level exceeded the baseline noise level. Thus, the onset of inhalation and exhalation corresponded to the EI transition and IE transition, respectively. The intraindividual RRI velocity was identified in each respiratory cycle. The series of cardiorespiratory parameters was synchronized with the series of cognitive parameters.

### Statistical analysis

Across individual subjects, we tested for normality and sphericity via the Shapiro-Wilk normality test and the Mauchly test for sphericity. We used one-way repeated-measures ANOVA, two-way ANOVA, *post hoc* pairwise comparisons via a paired *t* test with the Bonferroni correction, a two-tailed one-sample *t* test against zero, and Pearson’s product-moment correlation analysis. If the assumptions of normality and sphericity were violated, we performed the Wilcoxon rank sum test and nonparametric Friedman test followed by *post hoc* pairwise comparisons via the Wilcoxon signed-rank test with the Bonferroni correction and applied the Greenhouse-Geisser correction for departure from sphericity.

To further determine whether the duration of RT was modulated independently by respiratory timing (EI and IE phase transition), cardiac cycle extension, or performance accuracy, we used multilevel models that did not include other potential variables. The values of the mean RT, the appearance rates of the EI transition (EIt) and IE transition (IEt), the mean accuracy, and the mean RRI velocity were calculated every 2 task blocks (6 tests), with subtraction from the mean values of the intraindividuals after normalization among all the volunteers. We conducted multiple regression analyses with the mean RT as the dependent value. We separately set the appearance rates of EIt and IEt in the models because both rates were nonindependent components restricted in the respiratory cycle. While the appearance rates of EIt in the 2nd half, RRI velocity, and accuracy were used as independent values in Model 1, the appearance rates of IEt in the 2nd half, RRI velocity, and accuracy were used as independent values in Model 2.

$${\mathrm{RT}}_{\mathrm{ij}}={b_{0j}+b}_{1}{\left({{\mathrm{EIt}}^{2\mathrm{nd}}}_{\mathrm{ij}}-\overset{®}{{{\mathrm{EIt}}^{2\mathrm{nd}}}_{J}}\right)+b}_{2}(∆{\mathrm{RRI}}_{\mathrm{ij}}-\overset{®}{{∆\mathrm{RRI}}_{J}})+b_{3}({\mathrm{Accuracy}}_{\mathrm{ij}}-\overset{®}{{\mathrm{Accuracy}}_{J}})+r_{\mathrm{ij}}$$

Model 1 $${b_{0j}=\beta_{00}+s}_{0j}\;s_{0j}∼N(0,\;u^{2})\;r_{\mathrm{ij}}∼N(0,\;\sigma^{2})$$

$${\mathrm{RT}}_{\mathrm{ij}}={a_{0j}+a}_{1}{\left({{\mathrm{IEt}}^{2\mathrm{nd}}}_{\mathrm{ij}}-\overset{®}{{{\mathrm{IEt}}^{2\mathrm{nd}}}_{J}}\right)+a}_{2}(∆{\mathrm{RRI}}_{\mathrm{ij}}-\overset{®}{{∆\mathrm{RRI}}_{J}})+a_{3}({\mathrm{Accuracy}}_{\mathrm{ij}}-\overset{®}{{\mathrm{Accuracy}}_{J}})+z_{\mathrm{ij}}$$

Model 2 $$a_{0j}=\alpha_{00}+\;v_{0j}\;v_{0j}∼N(0,\;w^{2})\;z_{\mathrm{ij}}∼N(0,\;\gamma^{2})$$

RT_*ij*_ indicates the number *i* of RTs in subject *j*. Since there were individual differences (or different intercepts) in RT between the volunteers, we chose the random intercepts model. Notably, the regression coefficients (or slopes) were fixed between individuals. All the statistical analyses were performed via the lmerTest package in R version 3.6.1 software (R Core Team, R Foundation for Statistical Computing, Vienna, Austria, 2019, https://www.R-project.org/).

## Results

### RT extension according to the occurrence of the EI transition during the retrieval process

We investigated the functional involvement of the occurrence of respiratory phase transitions (i.e., IE and EI transitions) and RRI variability (i.e., RRI velocity) in task-related attentional processes. To do so, we examined accuracy and RT during a modified version of the DMTS task [19], [20] that thirty-six healthy participants performed. Each participant repeated three blocks of the DMTS task. In the task, each participant memorized four geometric images (sample cues in a sample section) and discriminated three geometric images (test cues in a test section) containing a symbol (configuration), its color, its position, and the number of instances of the symbol (Fig. 1a). A test trial (or retrieval process) was defined as the period from a test cue presentation to a button-press response. Although each test trial contained different degrees of lures, there were few differences in accuracy (Fig. 1b) and RT (Fig. 1c) in terms of the hit score (i.e., hit/hit + miss) and correct rejection score (i.e., correct rejection/correct rejection + false alarm) (accuracy in hit score: *χ*^2^ (2) = 5.80, *p* = 0.05495, Friedman test; accuracy in correct rejection: *χ*^2^ (2) = 12.49, *p* = 0.0019; RT in hit score: *χ*^2^ (2) = 2, *p* = 0.37; RT in correct rejection: *χ*^2^ (2) = 13.72, *p* = 0.0010; Lure 4: *p* = 0.03 to Lure 2). Each test trial was also classified into the following conditions (Fig. 1d): i) INH (inhalation) condition; ii) IEt (IE transition) condition; iii) EXH (exhalation) condition; iv) EIt (EI transition) condition; and v) double condition (test trials encompassing both the IE transition and EI transition, see Methods). The double condition was excluded because the number of test trials for each participant was too small. Although the number of INH condition was too small, the INH and EXH conditions were combined into the Not condition for further analysis. The presentation of the test cue was not intentionally aligned with the respiratory phase. Consequently, in the IEt and EIt conditions, IE and EI transitions incidentally occurred during the test trial. Moreover, respiratory rates and their coefficient of variation (CV) were adequate in the volunteers (Respiratory rate: 0.278 ± 0.008 Hz; CV: 26.80 ± 1.95 %).

As calculated by the Shapiro-Wilk normality test, accuracy was normally distributed among the participants, but RT was not. We found significant differences in total accuracy (n = 36 in each condition, *p* = 0.20 Mauchly test for sphericity; *F*(2, 70) = 4.37, *p* = 0.016, one-way repeated-measures ANOVA; Fig. 1f) and total RTs (*χ*^2^ (2) = 32.67, *p* = 8.1 × 10^−8^, Friedman test; Fig. 1g) among the conditions. *Post hoc* pairwise comparisons revealed that trials during the EIt condition had lower accuracy than those during the Not condition did (*p* = 0.029, paired *t* test with Bonferroni correction) and longer RTs than those during the Not and IEt conditions did (EIt: *p* = 0.0079 to IEt, *p* = 7.9 ×10^−8^ to Not, IEt: *p* = 1.7 ×10^−6^ to Not, Wilcoxon singed-rank test with Bonferroni correction). The results of the current study confirmed that respiratory transition-dependent effects (IEt and EIt conditions) increased RT in the DMTS task compared with that in the respiratory phase (INH and EXH conditions) and that in particular, the RT was longer in the EIt condition than in any other condition, as expected.

### Correct-trial RT increases when the EI transition occurs in the second half of the retrieval process

**Fig. 2:**
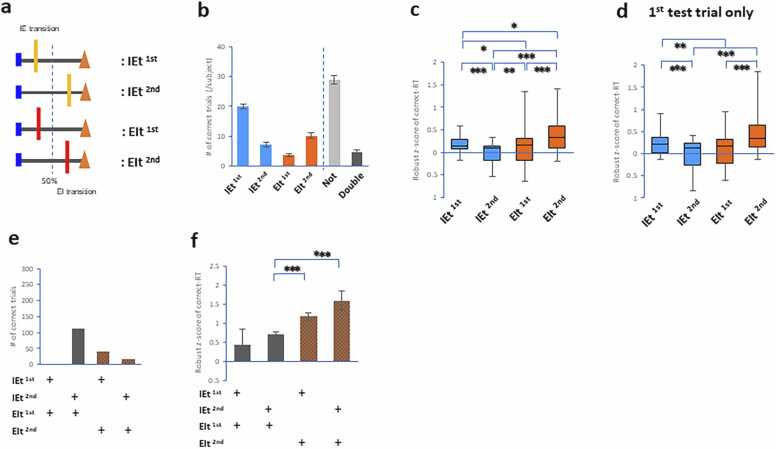
**RT in the occurrence of respiratory transitions during the retrieval process. a**. Drawing showing conditions of combinations between test trials and phases of the respiratory cycle: (i) test trials encompassing the IE transition in the 1st half of RT (IEt ^1st^); (ii) test trials encompassing the IE transition in the 2nd half of RT (IEt ^2nd^); (iii) test trials encompassing the EI transition in the 1st half of RT (EIt ^1st^); (iv) test trials encompassing the EI transition in the 2nd half of RT (EIt ^2nd^). **b-f**. Plots showing the number of correct test trials (**b,e**) and robust z scores of correct-trial RTs (**c,d,f**). The correct-trial RT was substantially extended when the EI transition occurred during the second half of the retrieval process. * p < 0.05, ** p < 0.01, *** p < 0.005 (post hoc comparisons).

Considering respiratory transition (IEt and EIt conditions)-dependent effects on RT, we further investigated whether their different stages altered RT, and we limited RT to correct test trials because an attentional process was strongly determined by longer RT with correct responses (e.g., attention network activity) [9]. The occurrence of EIt and IEt was divided into the 1st half and 2nd halves of the correct-trial RT, respectively: i) the IEt ^1st^ condition; ii) the IEt ^2nd^ condition; iii) the EIt ^1st^ condition; and iv) the EIt ^2nd^ condition (Fig. 2a; see Methods). RT was standardized by the median and interquartile range in individual subjects. The standardized RT (i.e., robust z score) for these conditions was averaged.

Using two datasets from our previous studies (Nakamura et al., 2018, 2022), we first conducted detailed analyses of correct-trial RT when the respiratory transitions (IEt and EIt conditions) occurred in two separate stages during test trials of the DMTS task, which included the memorization of four geometric images (sample cues in a sample section) and the discrimination of 10 geometric images (test cues in a test section, Suppl. Fig. S1). These datasets confirmed that the occurrence of the EIt ^2nd^ condition presented a longer RT limited to correct responses than any other condition did.

Next, the current study confirmed whether the correct-trial RT was extended when the EI transition occurred in the second-half stage during three test trials of the DMTS task. The Shapiro-Wilk normality test revealed that correct-trial RTs were not normally distributed. The Friedman test revealed a significant difference in correct-trial RTs across the conditions (n = 36 in each condition; *χ*^2^ (3) = 42.53, *p* = 3.1 × 10^−9^; Fig. 2b, c). *Post hoc* pairwise comparisons revealed that the EIt ^2nd^ condition had the longest RT limited to correct responses among all the conditions (*p* = 0.029 to IEt ^1st^, *p* = 9.6 ×10^−9^ to IEt ^2nd^, *p* = 0.00047 to EIt ^1st^; Wilcoxon singed-rank test with Bonferroni correction). Furthermore, the EIt ^2nd^ condition also had a longer RT limited to correct responses when we restricted it to the first test trial of the DMTS task (*χ*^2^ (3) = 53.76, *p* = 1.3 × 10^−11^, Kruskal-Wallis rank sum test; EIt ^2nd^: *p* = 5.9 × 10^−12^ to IEt ^2nd^, *p* = 4.0 × 10^−5^ to EIt ^1st^; IEt ^1st^: *p* = 0.0074 to EIt ^1st^, *p* = 7.9 × 10^−9^ to EIt ^1st^, pairwise comparisons using the Wilcoxon rank sum exact test, Fig. 2d). We revealed that the correct-trial RT substantially increased when the EI transition occurred during the second half of the retrieval process.

Although the Double condition encompassed both the EIt and IEt, it is possible to identify distinct effects within the Double condition by using divisions of the occurrence of respiratory transitions between the 1st half and 2nd halves of the RT during test trials. Since the number of participants in the Double condition was smaller (n = 10 subjects), we divided individual test trials in the Double condition into four additional conditions: i) the IEt ^1st^ and EIt ^1st^ conditions; ii) the IEt ^2nd^ and EIt ^1st^ conditions; iii) the IEt ^1st^ and EIt ^2nd^ conditions; and iv) the IEt ^2nd^ and EIt ^2nd^ conditions (Fig. 2e). We used the standardized RT of individual test trials among the conditions for further analysis. The correct-trial RTs were normally distributed. Two-way ANOVA revealed significant main effects of IEt and EIt (IEt: *F*(1, 167) = 8.22, *p* = 0.0047; EIt: *F*(1, 167) = 33.28, *p* = 3.8 × 10^−8^; Fig. 2f). *Post hoc* pairwise comparisons revealed that the EIt ^2nd^ component increased the correct-trial RT compared with the EIt ^1st^ component (both IEt ^2nd^ and EIt ^2nd^: *p* = 7.0 ×10^−7^ compared with both IEt ^2nd^ and EIt ^1st^; both IEt ^1st^ and EIt ^2nd^: *p* = 0.00038 compared with both IEt ^2nd^ and EIt ^1st^, *t* tests with pooled standard deviation with Bonferroni correction). These results showed that the occurrence of the EIt in the second half of the retrieval process induced the extension of correct-trial RTs in the Double condition.

### Correct RT extension by the high positive velocity of the RRI during the retrieval process

**Fig. 3:**
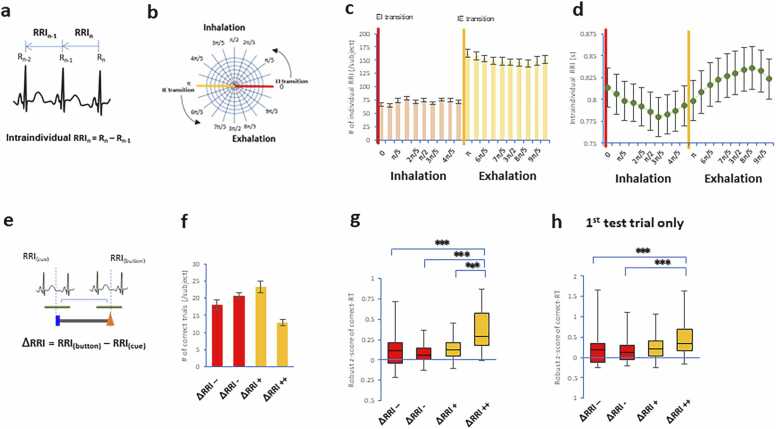
**RT in terms of changes in RRI velocity during the retrieval process. a**. Drawing showing electrocardiograms. The intraindividual RRIs were measured from an R wave (Rn) to the previous R wave (Rn-1). **b-d**. Plots showing the number of intraindividual RRIs (**c**) and intraindividual RRIs (**d**) during inhalation and exhalation in the respiratory cycle. **e**. Drawing showing conditions of the ΔRRI between test trials and intraindividual RRIs. The ΔRRI was calculated by subtracting the RRI overlapping a test cue presentation from the RRI overlapping a button-press response. **f-h**, Plots showing the number of correct test trials (**f**) and robust z scores of correct-trial RTs (**g,h**). The correct-trial RTs were extended by high positive RRI velocities during the retrieval process of the task. *** p < 0.005 (post hoc comparisons).

Generally, intraindividual RRIs are calculated by the reciprocal of intraindividual heart rates and fluctuate during respiratory phases. The intraindividual RRIs decreased during inhalation and increased during exhalation in thirty-six volunteers during the DMTS task (Fig. 3a-d). To determine whether the correct-trial RT is altered by cardiac variability during the retrieval process of the task, we defined the RRI velocity (ΔRRI) during the test trials as follows: the ΔRRI was calculated by subtracting the RRI overlapping a test cue presentation from the RRI overlapping a button-press response (Fig. 3e).

To account for the multiplicative nature of velocity changes and to stabilize variance across conditions, RRI velocity values were analyzed in log-transformed space. This transformation allows proportional (e.g. twofold) changes in speed to be represented linearly. We then divided the RRI velocity into four conditions according to the proportion of maximum RRI velocity in individual volunteers: i) high negative RRI velocity (-1 to log_10_(0.5) or 30–100 % negative rate, ΔRRI --); ii) low negative RRI velocity (log_10_(0.5) to 0 or 0–30 % negative rate, ΔRRI -); iii) low positive RRI velocity (0 to log_10_(2) or 0–30 % positive rate, ΔRRI + ); and iv) high positive RRI velocity (log_10_(2) to 1 or 30–100 % positive rate, ΔRRI + +) (Fig. 3f). With respect to RT, the robust z score of correct-trial RTs was standardized by the median and interquartile range in individual subjects. The standardized RT was subsequently averaged for each condition within each subject. The Shapiro-Wilk test revealed that the correct-trial RTs for the four RRI velocity conditions were not normally distributed. The Friedman test revealed significant differences in correct-trial RTs across the conditions (n = 36 in each condition; χ^2^ (3) = 32.73, p = 3.7 × 10^−7^, Fig. 3g). *Post hoc* pairwise comparisons revealed that high positive RRI velocity (ΔRRI ++) had the longest RT in trials with correct responses compared with all the conditions of RRI velocity (*p* = 0.00034 to ΔRRI --, *p* = 3.8 ×10^−6^ to ΔRRI -, *p* = 1.2 ×10^−6^ to ΔRRI +, Wilcoxon singed-rank test with Bonferroni correction). In addition, the high positive RRI velocity (ΔRRI ++) also had a longer RT limited to correct responses when we restricted it to the first test trial of the DMTS task (*χ*^2^ (3) = 20.03, *p* = 0.000167, Kruskal-Wallis rank sum test; ΔRRI + +: *p* = 0.0012 to ΔRRI --, *p* = 0.00078 to ΔRRI -, pairwise comparisons using the Wilcoxon rank sum exact test, Fig. 3h). Moreover, we achieved similar results with another RRI velocity (i.e., ΔRRI_b_) dataset, where the RRI at a state before a test cue presentation was subtracted from the RRI overlapping a button-press response (Suppl. Fig. S2). These results showed that correct-trial RTs were extended by high positive RRI velocities during the retrieval process of the task.

### Multilevel model analyses

To further elucidate the distinct effects of respiratory phase transition and RRI variability on attentional processing, we conducted multilevel regression analysis of Model 1 with RT as the dependent variable and the occurrence rates of EIt in the 2nd half (EIt ^2nd^), RRI velocity (ΔRRI), and accuracy as independent variables (Table 1). Notably, we separated the occurrence rates of EIt and IEt in the models because both rates are nonindependent components restricted to the respiratory cycle. In Model 1, the results revealed that RT was significantly positively correlated with the occurrence rate of EIt in the 2nd half and the RRI velocity, whereas RT was significantly negatively correlated with accuracy. Moreover, in Model 2, we used the occurrence rate of IEt in the 2nd half (IEt ^2nd^) as an independent variable instead of the occurrence rate of EIt in the 2nd half (EIt ^2nd^). This model failed to show a significant correlation coefficient for IEt (Table 2). However, RT maintained a positive correlation with the RRI velocity and a negative correlation with accuracy. These results indicated that changes in RT were distinct between the occurrence of EIt in the 2nd half and the RRI velocity, whereas the RRI velocity had a positive effect on RT independent of IEt.

**Table 1:** Regression coefficients of the multilevel model with random intercept (Model 1).

	regression coefficient (SE)	p-value
EIt^2nd^	0.210 (0.0334)	6.42 × 10^−10^
ΔRRI	0.145 (0.0401)	3.21 × 10^−4^
Accuracy	-0.118 (0.0310)	1.46 × 10^−4^

**Table 2:** **Regression coefficients of the multilevel model with random intercept (Model 2)**.

	regression coefficient (SE)	p-value
IEt^2nd^	-0.0188 (0.0317)	0.553
ΔRRI	0.0934 (0.0411)	0.0235
Accuracy	-0.130 (0.0321)	5.94 × 10^−5^

### Relationship between the occurrence of EIt and increase in RRI velocity

**Fig. 4:**
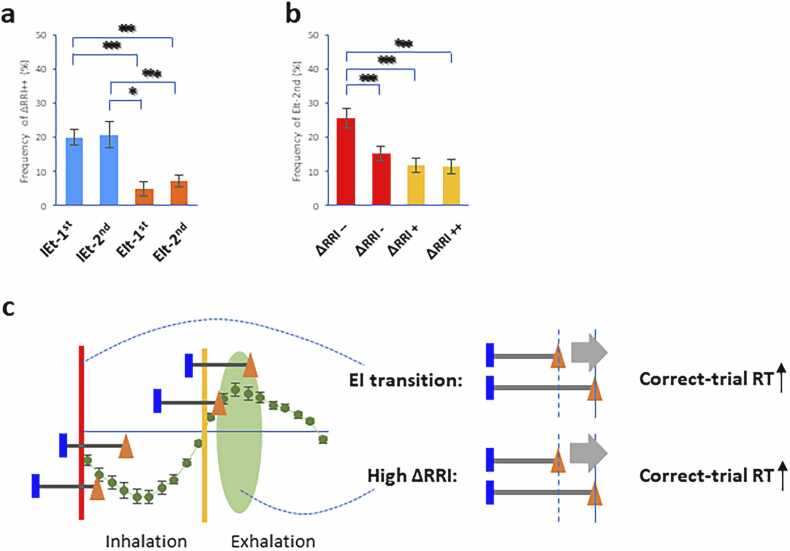
**Frequency of test trials between the increase in RRI velocity and occurrence of EIt**^**2nd**^**. a,b**. Plots showing the frequency of the increase in RRI velocity during the respiratory transition (**a**) and the frequency of the occurrence of EIt 2nd in terms of RRI velocity (**b**). * p < 0.05, *** p < 0.005 (post hoc comparisons). **c**. Schematic illustrating that occurrences of the EIt-2nd and high positive RRI velocities are associated with increased correct-trial RT, potentially via functionally distinct attentional processes.

Although multilevel regression analysis demonstrated that RT was characterized separately between the occurrence of the EI transition and the increase in RRI velocity, the temporal effects of RT on the retrieval process remain to be determined. To understand their peak frequency during the test trials, we analyzed the frequency distribution of high positive ΔRRIs in the respiratory cycle and the frequency distribution of the occurrence of EIt-2nd in terms of changes in RRI velocity (also see Suppl. Fig. S3). The Shapiro-Wilk test showed normality in the frequency of high positive RRI velocity and the frequency of the occurrence of EIt-2nd. One-way repeated-measures ANOVA revealed a significant difference in the frequency rate of high positive RRI velocity (n = 36 in each condition, *p* = 2.0 ×10^−6^, Mauchly test for sphericity; *F*(3, 105) = 12.48, *p* = 4.3 × 10^−5^ with Greenhouse-Geisser correction for departure from sphericity, Fig. 4a). *Post hoc* pairwise comparisons revealed that the IEt-1st and IEt-2nd conditions had higher frequency rates of high positive RRI velocities than the EIt-1st and EIt-2nd conditions did (IEt-1st: *p* = 7.6 ×10^−5^ to EIt-1st, *p* = 0.00098 to EIt-2nd, IEt-2nd: *p* = 0.0022 to EIt-1st; *p* = 0.033 to EIt-2nd, paired t test with Bonferroni correction). Moreover, there was a significant difference in the frequency rate of EIt-2nd (n = 36 in each condition, *p* = 0.011, Mauchly test for sphericity; *F*(3, 105) = 11.03, *p* = 2.7 × 10^−5^ with Greenhouse-Geisser correction for departure from sphericity, Fig. 4b). *Post hoc* pairwise comparisons revealed that a high negative RRI velocity had a greater frequency rate of EIt-2nd than the other conditions did (*p* = 0.00093 for a low negative ΔRRI, *p* = 0.0011 for a low positive ΔRRI, *p* = 0.0013 for a high positive ΔRRI, paired *t* test with Bonferroni correction). These results revealed that the peak frequency of occurrence between EIt-2nd and high positive RRI velocity was temporally divided during the test trials.

## Discussion

The present study demonstrated that the occurrence of inspiratory onset (EIt) and RRI variability were critical factors that increased RTs in the DMTS task. Because attentional processes are strongly reflected in longer RTs with successful performance (e.g., attention network activity), we preferentially analyzed RTs limited to correct responses that were prolonged under two conditions: i) when the EIt occurred in the 2nd half of the retrieval phase (Fig. 2) and ii) when the RRI velocity increased during the retrieval phase (Fig. 3). Importantly, multilevel model analysis revealed that the occurrence of EIt and the increase in RRI velocity independently contributed to longer RTs (Table 1), suggesting that at least these two attentional mechanisms may modulate RTs. Moreover, there was no significant effect of IEt occurrence on the prolongation of RT, whereas the RRI velocity again contributed to RT extension (Table 2), indicating that RRI fluctuations directly influence RT. Since the EIt and increase in RRI velocity were temporally misaligned during retrieval (Fig. 4), inspiratory onset and RRI fluctuations may arise from partially distinct mechanisms that might be related to different attentional processes affecting task performance.

RSA is considered an essential index of cardiac vagal tone that may reflect attentional states [34], [35], even though estimation of the index has not yet been elucidated [38], [39]. RSA may be linked to autonomic nervous system (ANS) activity via the pontomedullary network, which induces fluctuations in RRIs [40]; specifically, sympathetic activity reflects short RRIs during inhalation, and parasympathetic activity reflects long RRIs during exhalation (postinspiration and active expiration) in humans [37], [41]. Thus, a decreased RRI velocity can be sympathetically dependent, and an increased RRI velocity can be parasympathetically dependent. In particular, the frequency of RSA is closely related to the power spectrum of heart rate variability (HRV), especially the high-frequency (HF) power (0.15–0.4 Hz, HF-HRV). HRV is utilized to determine an aspect of ANS activity, and its biofeedback application is used for therapeutic effects and improvement of autonomic homeostasis [42]. However, cognitive recruitment results in a higher rate of sighs and incoherence of respiratory waveforms [20], [43], [44]. Practically, sighs contribute to causing slower variations in RRIs, resulting in the power during the sigh phase shifting into the low-frequency band (0.04–0.15 Hz) in the spectrum (data not shown). Moreover, mental stress induced incoherent oscillations of RSA with respect to the breathing rate [45]. Thus, it is likely that estimation of RSA requires another dimension of heart rate activity, such as the ratio of phase synchronization between the breathing cycle and RRI fluctuations [45], [46], individual values of RSA amplitude [37], and individual values of RRI velocity that we applied.

We found that the occurrence of inspiratory onset (EIt) and an increase in RRI velocity (ΔRRI) independently prolonged RTs during task-related attentional processes. Because the temporal interval between EIt and high ΔRRI spans at least the duration of the inspiratory phase (i.e., > 1 sec), these events are temporally mismatched during retrieval, suggesting that at least two distinct mechanisms may contribute to RT prolongation. Our previous study demonstrated that the occurrence of EIt during retrieval extended RTs, reduced accuracy, and deactivated the right anterior part of the temporoparietal junction (TPJ), a neural hub of the VAN in healthy volunteers performing the DMTS task, as shown by fMRI[20]. Indeed, EIt occurrence was associated with ∼90 % accuracy in test trials and deactivation of the VAN, indicating the engagement of a specific form of attention [21].

The present findings showed that increased RRI velocity prolonged RTs exclusively in correct responses, suggesting engagement of a distinct attentional mechanism. Because the increase in RRI velocity predominantly occurred during exhalation, it may reflect parasympathetic dominance. Parasympathetic-supporting neural hubs are the NTS and the anterior insula [22], [23], [47.]. The anterior insula has preferential connections to the dorsal anterior cingulate cortex (dorsal ACC) and ventromedial prefrontal cortex (also known as the pregenual and subgenual ACC) [48], [49], [50], regions essential for visceromotor autonomic regulation [51], [52]. Together, these structures contribute to the central autonomic network [53], [54], [55] and salience network [56.], [57.], [58.], where cardiovascular signals are integrated to modulate autonomic activity [47]. In contrast, EI transition likely suppresses attentional processes via transient deactivation of the TPJ, a key node of the VAN [20]. The DAN, anchored in the intraparietal sulcus, lies in close spatial proximity to the TPJ [59]. This adjacency is thought to constitute a connector or “shifting zone” that enables rapid interactions between stimulus-driven (VAN) and goal-directed (DAN) attentional processes [60]. Such an arrangement suggests that VAN-DAN interactions may transiently amplify top-down orienting signals, thereby prolonging RT when retrieval is interrupted at EI transition. Further neuroimaging research will be essential to further delineate how inspiratory onset and RRI velocity independently engage these attention-related networks.

A sample size of 36 participants provides > 80 % statistical power to detect effects of the magnitude in a within-subject design. Our own prior work using a comparable DMTS paradigm also demonstrated robust phase-dependent effects with similar effect sizes and stable within-individual variance structures [20]. Thus, the current sample is well powered for detecting the hypothesized differences in RT and accuracy.

Intraindividual RT variability may be linked to deficits in attention during a task [61], [62] and is considered to reflect an etiologically important characteristic of attention-deficit hyperactivity disorder [63]. RT variability refers to inconsistency in an individual’s response speed, measured in seconds or milliseconds, and has been suggested to reflect a subset of abnormally slow responses during tasks. Importantly, previous studies have shown that intraindividual RT variability is negatively correlated with HF-HRV during not only a resting state [64] but also a visual attentional task [65]. However, since brain network activity differs between tasks with shorter and longer RTs [8], [9], analyzing RTs separately is appropriate to better understand the distinct effects of respiration and RRI variability.

In conclusion, our results identify two discrete points within the respiratory cycle at which attentional processing is altered, implicating mechanistically distinct pathways. One aligns with the EI transition (inspiratory onset), likely reflecting direct gating by medullary respiratory drive, whereas the other appears to arise from parasympathetically mediated regulation in attentional engagement indexed by RRI variability. These findings highlight the critical role of respiratory timing in sustaining successful cognitive performance and point to future applications aimed at optimizing task demands through real-time visualization of interoceptive rhythms.

## CRediT authorship contribution statement

**Masaki Fukunaga:** Writing – review & editing, Methodology, Data curation. **Kohzoh Yoshino:** Writing – review & editing, Validation, Software, Resources, Methodology, Formal analysis. **Nakamura Nozomu:** Writing – review & editing, Writing – original draft, Visualization, Validation, Supervision, Software, Resources, Project administration, Methodology, Investigation, Funding acquisition, Formal analysis, Data curation, Conceptualization.

## Author contributions

NHN contributed to the study design; NHN contributed to acquisition of data; NHN and MF contributed to methodology; NHN and KY contributed to data analysis; NHN and KY contributed to data interpretation; NHN contributed to drafting of manuscript; NHN, KY and MF contributed to the final manuscript.

## Declaration of Competing Interest

The authors declare that they have no known competing financial interests or personal relationships that could have appeared to influence the work reported in this paper.

## Data Availability

The data that support the main findings of this study are available from the corresponding author upon request. The datasets used in this study are also available at https://github.com/nakamunh/Nakamura_2025_JPS.

## Footnotes

Appendix A Supplementary data associated with this article can be found in the online version at doi:10.1016/j.jphyss.2025.100053.
