# Supplementary material for: Task-related attentional processes are distinctly modulated by respiration and RR interval variability
Source: J Physiol Sci. 2025 Dec 22;76(1):100053. doi: 10.1016/j.jphyss.2025.100053 (PMC12803905; doi:10.1016/j.jphyss.2025.100053)
Supplement: Supplementary file 1 — Supplementary material [file mmc1.pdf]

Fig. S1

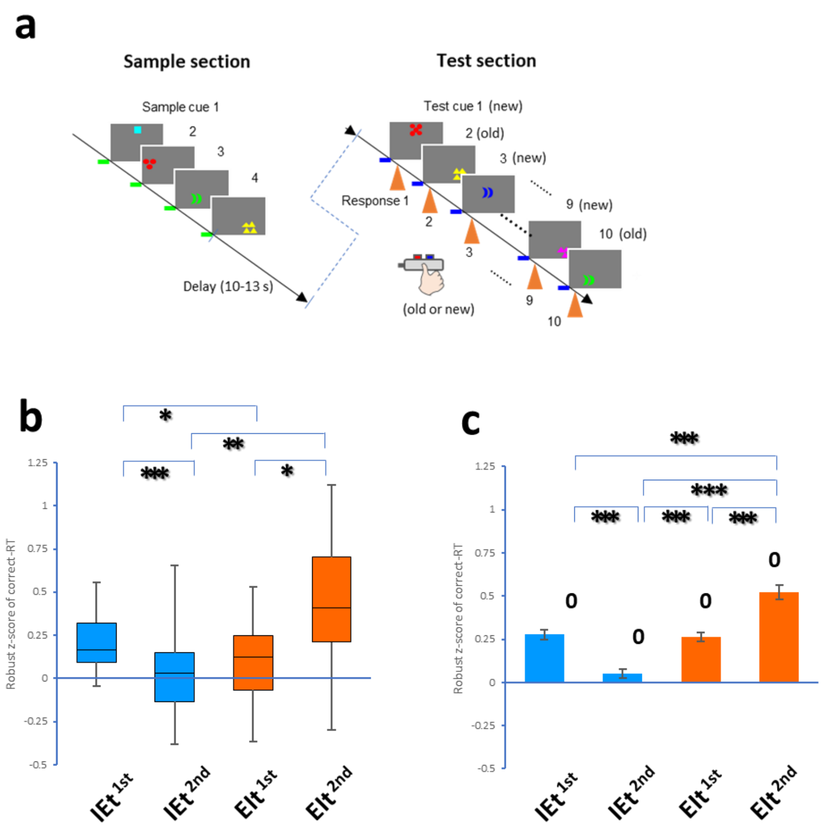


**Fig. S1. RT in the occurrence of respiratory transitions in our previous datasets**

In our previous studies (Nakamura et al., 2018, 2022), healthy volunteers memorized 4 sample cues and discriminated 10 test cues (**a**). Appearance of EI and IE transitions was divided into 1$^{st}$ half and 2$^{nd}$ half of RT durations limited with correct answers, and the standardized RT was averaged within each subject. While the dataset from Nakamura et al. (2018) did not have normality of distribution, the dataset from Nakamura et al. (2022) had normality. Regarding the dataset from Nakamura et al. (2018), Friedman test showed a significant difference in correct-trial RT in Nakamura et al. (2018; n = 18 in each condition; $\chi^2$ (3) = 18.8, p = 0.00030, **b**). *Post hoc* pairwise comparisons showed that EIt-2$^{nd}$ condition had longer correct-trial RT than IEt-2$^{nd}$ and EIt-1$^{st}$ conditions ($p$ = 0.0063 to IEt-2$^{nd}$, $p$ = 0.033 to EIt-1$^{st}$; Wilcoxon singed rank test with Bonferroni correction). Furthermore, IEt-1$^{st}$ had longer correct-trial RT than IEt-2$^{nd}$ and EIt-1$^{st}$ conditions (p = 0.0032 to IEt-2$^{nd}$, p = 0.029 to EIt-1$^{st}$). Regarding the dataset in Nakamura et al. (2022), one-way repeated-measures ANOVA showed a significant difference in correct-trial RT (n = 25 in each condition, p = 0.63, Mauchly test for sphericity; F(3, 72) = 46.80, p = 2.2 x $10^{-16}$, **c**). *Post hoc* pairwise comparisons showed that EIt-2$^{nd}$ condition had the longest correct-trial RT than all the conditions (p = 7.1 x $10^{-5}$ to IEt-1$^{st}$, $p$ = 1.5 x $10^{-9}$ to IEt-2$^{nd}$, $p$ = 1.3 x $10^{-5}$ to EIt-1$^{st}$; paired t test with Bonferroni correction). These previous datasets showed that EI transition at the second half of the retrieval process extended RT limited to correct responses.

Fig. S2

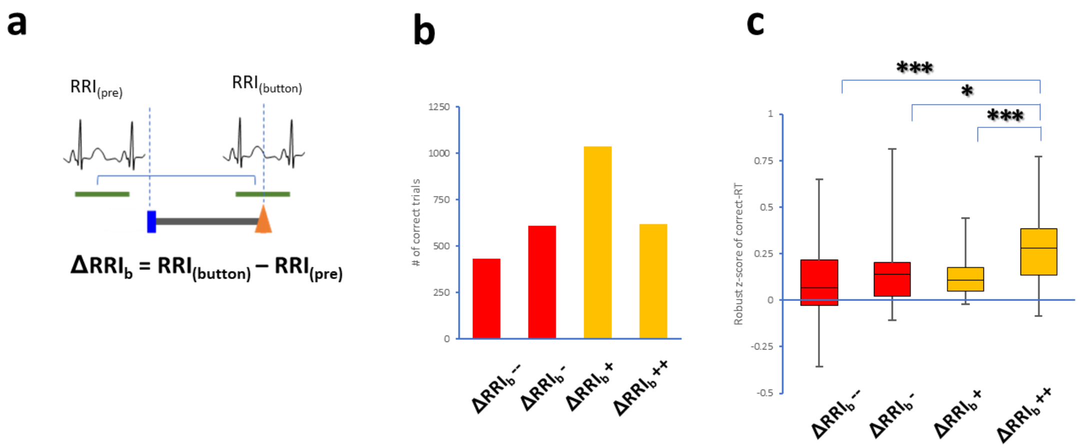


**Fig. S2. A different set of RT in changes in RRI velocity during the retrieval process**

To determine RRI velocity during RT in the task, we calculated with another value of RRI velocity ($\Delta RRI_b$), where RRI at a state before a test cue presentation is subtracted RRI overlapping a button-press response. The Shapiro-Wilk test did not show normality in correct-trial RT for the four conditions of RRI velocity. Then, Friedman test showed significant differences in correct-trial RT among the conditions (n = 36 in each condition; $\Delta RRI_b$: $\chi^2$ (3) = 12.63, p = 0.0055). *Post hoc* pairwise comparisons showed that high positive RRI velocity (ΔRRI ++) had the longest RT with correct responses in all the conditions of RRI velocity ($\Delta RRI_b$: $p$ = 0.0025 to ΔRRI --, $p$ = 0.019 to ΔRRI -, $p$ = 0.0012 to ΔRRI +, Wilcoxon singed rank test with Bonferroni correction). These results showed that correct-trial RT was prolonged by high positive velocity of RRI during the retrieval process of the task.

Fig. S3

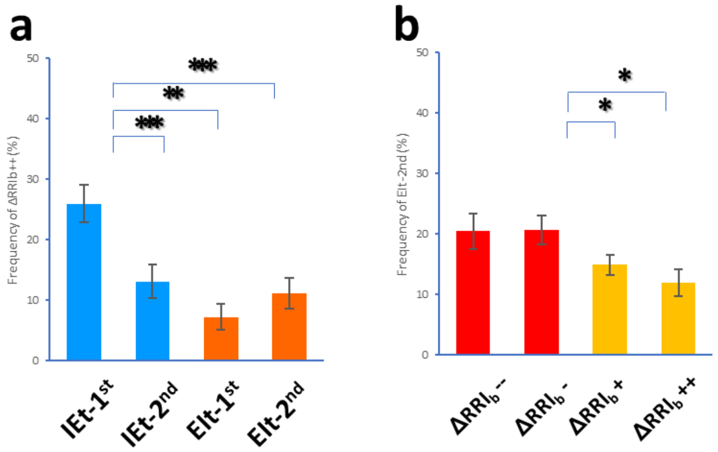


**Fig. S3. A different set of frequency of test trials between the increase of RRI velocity and occurrence of EIt $^{2nd}$**

The Shapiro-Wilk test showed normality in appearance ratio of high positive RRI velocity (**a**) and appearance ratio of EIt-2$^{nd}$ (**b**) regarding the four conditions, respectively. One-way repeated-measures ANOVA showed a significant difference in appearance ratio of high positive RRI velocity ($\Delta RRI_b$, n = 36 in each condition, $p$ = 0.00030, Mauchly test for sphericity; $F(3, 105) = 4.93$, $p$ = 0.0072 with Greenhouse-Geisser correction for departure from sphericity, **a**). *Post hoc* pairwise comparisons showed that IEt-1$^{st}$ condition had higher appearance ratio of high positive RRI velocity than all the conditions ($p$ = 0.0054 to IEt-2$^{nd}$, $p = 2.0 \times 10^{-5}$ to EIt-1$^{st}$, $p$ = 0.0048 to EIt-2$^{nd}$, paired t test with Bonferroni correction). There was a significant difference in appearance ratio of EIt-2$^{nd}$ (n = 36 in each condition, $p$ = 0.31, Mauchly test for sphericity; $F(3, 105) = 10.94$, $p = 2.6 \times 10^{-6}$, **b**). *Post hoc* pairwise comparisons showed that low negative RRI velocity had higher appearance ratio of EIt-2$^{nd}$ than positive $\Delta RRI_b$ conditions ($p$ = 0.014 to low positive $\Delta RRI_b$, $p$ = 0.027 to high positive $\Delta RRI_b$, paired t test with Bonferroni correction).
